# ABCC6 Heterozygosity as Genetic Predisposition to Cerebrovascular Disease Across Ages

**DOI:** 10.3390/genes17020226

**Published:** 2026-02-11

**Authors:** Giulia Amico, Mariasavina Severino, Marta Bertamino, Rosario Pascarella, Domenico Tortora, Sara Signa, Marta Rusmini, Andrea Rossi, Isabella Ceccherini, Marialuisa Zedde

**Affiliations:** 1 Laboratory of Human Genetics, IRCCS Istituto Giannina Gaslini, Via Gerolamo Gaslini, 5, 16147 Genoa, Italy; 2 Gaslini Stroke Study Groupisabellaceccherini@gaslini.org (I.C.); 3 Neuroradiology Unit, IRCCS Istituto Giannina Gaslini, Via Gerolamo Gaslini, 5, 16147 Genoa, Italy; 4 Physical Medicine and Rehabilitation Unit, IRCCS Istituto Giannina Gaslini, Via Gerolamo Gaslini, 5, 16147 Genoa, Italy; 5 Neuroradiology Unit, Ospedale Santa Maria della Misericordia, AULSS5 Polesana, 45100 Rovigo, Italy; 6 Rare Diseases Unit, IRCCS Istituto Giannina Gaslini, Via Gerolamo Gaslini, 5, 16147 Genoa, Italy; 7 Genetics and Genomics of Rare Diseases Unit, IRCCS Istituto Giannina Gaslini, Via Gerolamo Gaslini, 5, 16147 Genoa, Italy; 8 Clinic Bioinformatics Unit, IRCCS Istituto Giannina Gaslini, 16100 Genoa, Italy; 9 Medical Genetics Unit, IRCCS Istituto Giannina Gaslini, Via Gerolamo Gaslini, 5, 16147 Genoa, Italy; 10 Neurology Unit, Stroke Unit, Azienda Unità Sanitaria Locale, IRCCS di Reggio Emilia, 42123 Reggio Emilia, Italy

**Keywords:** *ABCC6*, stroke, pediatric, monoallelic, intracranial stenosis, GACE, pseudoxanthoma elasticum

## Abstract

**Background**: Heterozygosity for pathogenic variants in the *ABCC6* gene has been associated with an increased incidence of cerebrovascular diseases. This study aims to characterize the prevalence and clinical and neuroradiological phenotypes associated with monoallelic and biallelic ABCC6 variants in pediatric and adult patients presenting with arterial ischemic stroke or cerebral small vessel disease (CSVD). **Methods**: We conducted a retrospective observational study on 143 consecutive patients (48 pediatric, 24 juvenile, 71 adult) diagnosed with ischemic stroke or CSVD of unknown etiology. Clinical and neuroradiological data were collected and analyzed in relation to the identified genetic variants through next-generation sequencing. **Results**: Among the patients, 16 (11.2%) tested positive for causative variants in the *ABCC6* gene, with 11 subjects carrying monoallelic variants and 5 carrying biallelic variants. Patients with biallelic variants exhibited severe and complex vasculopathy, with a high incidence of early ischemic events. In contrast, monoallelic carriers predominantly presented with microvascular disease manifestations, including lacunar strokes and signs of CSVD. **Conclusions**: The results suggest a significant age-dependent phenotypic divergence in patients with ABCC6 variants, highlighting the impact of heterozygosity on cerebrovascular health. Identifying these variants may enhance risk stratification and inform management strategies in patients with traditional vascular risk factors.

## 1. Introduction

The ATP-binding cassette subfamily C member 6 (*ABCC6*) transporter, encoded by the homonymous gene, facilitates the ATP efflux that, whenever hydrolyzed into inorganic pyrophosphate (PPi), contributes to the physiological homeostasis of the mineralization process. ABCC6 biallelic causative variants are associated with generalized arterial calcification of infancy (GACI-OMIM #614473) and classical pseudoxanthoma elasticum (cPXE–OMIM #264800), both inherited through an autosomal recessive fashion. Both disorders share the same underlying disease mechanism based on ectopic mineralization leading to calcification and fragmentation of the elastic fibers, especially in the eye, skin, and blood vessels [1]. Consequent pathognomonic features of GACI and cPXE are papular cutaneous lesions, skin laxity, angioid retinal streaks, choroidal neovascularization and hemorrhage, systemic and intracranial arteriopathy, ischemic and hemorrhagic stroke, coronary artery calcification, and myocardial infarction [1]. Since no genotype–phenotype correlation has been reported so far, the clinical and neuroradiological features remain key to distinguish cPXE from GACI [1,2,3,4,5]. This last assumption particularly applies to the pediatric age group. For adults, GACI is not a differential diagnosis, and the arterial calcifications as a pathognomonic element diminish significantly, as there are many other common conditions that can determine and justify it compared to children.

Although debated in the past literature due to the relatively high carrier frequency of *ABCC6* pathogenic variants in the general population (~1%) [6,7,8,9], heterozygous individuals seem to display a marked milder form of cPXE defined as forme fruste PXE (ffPXE—OMIM #177850). These individuals do not display all the complete and progressive signs of cPXE but only some less severe features of the disease, including retinal manifestations, cutaneous lesions, altered lipidic metabolism, ectopic calcification, and coronary artery disease [10,11,12,13,14,15,16,17]. Regarding the association with cerebrovascular disease, in 2018 De Vilder et al. [12] demonstrated that monoallelic pathogenic *ABCC6* variants were 4.9 times more frequent in a young adult/adult ischemic stroke cohort compared with healthy controls, suggesting *ABCC6* partial deficiency/disfunction as a cerebrovascular risk factor. In this study, the stroke phenotypes were diverse, mainly including large vessel (LV) disease but also cerebral small vessel disease (CSVD) and cardioembolic stroke [12]. More recently, Cho et al. reported heterozygous *ABCC6* variants in adult subjects with suspected familial cerebral SVD [18] and Zedde and Pascarella [19] deepened the potential mechanisms and associations. However, available information on adulthood is very limited and is affected by individual and small cohorts with a heterogeneity of potentially confounding factors. Indeed, the neuroradiological phenotype of CSVD, common in adulthood, is multifactorial and often influenced by the simultaneous presence of classic vascular risk factors and unhealthy lifestyles, in addition to genetic issues. Different cohorts than those selected for ischemic stroke as the index event are not described, thus limiting information on the non-ischemic presentation and neuroradiological patterns of CSVD in the absence of ischemic stroke, as well as on large cohorts of patients with spontaneous arterial dissection with or without stroke. Finally, data in the literature are still scarce and no information regarding the prevalence and clinical and radiological manifestations of heterozygous *ABCC6* pathogenic variants in the pediatric and adult stroke populations are available.

In this retrospective observational study, we assess the prevalence of monoallelic pathogenic *ABCC6* variants in a cohort of consecutive pediatric, juvenile, and adult patients with arterial ischemic stroke or cerebral small vessel disease. We also characterize the clinical and neuroradiological features of heterozygous carriers, comparing pediatric and adult subgroups and contrasting their findings with those observed in patients carrying biallelic mutations associated with recessive *ABCC6* disorders (cPXE and GACI).

## 2. Materials and Methods

### 2.1. Ethical Considerations

This retrospective observational study was conducted in accordance with ethical standards laid down in the 1964 Declaration of Helsinki and with more recent recommendations on genetic testing (American Academy of Pediatrics Committee on Bioethics, Committee on Genetics, American College of Medical Genetics, and Genomics Social Ethical and Legal Issues Committee, 2013) [20], as well as on the evaluation and validation of NGS applications for the diagnosis of genetic disorders [21]. The study was also approved by the Gaslini Ethical Committee (n. 096/2019). Written informed consent was obtained from patients >18 years and from parents on behalf of patients < 18 years.

### 2.2. Selection of the Cohort

In this study, we enrolled all consecutive patients with pediatric or juvenile/adult cerebral vasculopathy with or without arterial ischemic stroke or SVD of unknown etiology, referred for diagnostic workup and second opinion to two Italian pediatric and/or adult stroke centers (IRCCS Istituto Giannina Gaslini in Genoa and IRCCS Azienda Unità Sanitaria Locale in Reggio Emilia) between July 2014 and June 2024. Patients were included after a genetic diagnosis of *ABCC6* mutation without mutations of the other genes present in a wide genetic panel routinely performed for cerebrovascular diseases in the Genetics Laboratory at the IRCCS Istituto Giannina Gaslini. The access to the genetic test was proposed if they presented one of the following clinical–radiological phenotypes related to pediatric or juvenile/adult ischemic stroke or CSVD with a potential underlying genetic cause: (i) AIS with cerebral arteriopathy and/or multi-system arteriopathy; (ii) multiple recurrent AIS even without cerebral arteriopathy; (iii) AIS in syndromic patients and/or family history of cerebro-cardiovascular disorders; (iv) CSVD with a positive family history; (v) spontaneous arterial dissection; (vi) family members with PXE or GACI. Exclusion criteria included the following: (i) previous intracranial radiotherapy; (ii) underlying conditions known to be risk factors for stroke in children and adults; (iii) concomitant or recent infection (such as varicella zoster virus) [22,23]; (iv) major head–neck trauma; (v) chromosomal aberrations predisposing to vasculopathy (Down syndrome, Turner syndrome) [24]; (vi) inflammatory focal cerebral arteriopathy; (vii) fibromuscular dysplasia showing a prevalent extracranial involvement; (viii) previous genetic diagnosis correlated with the vascular phenotype (such as CADASIL, CARASIL). Due to their high population frequency, patent foramen ovale and minor thrombophilia were not considered as exclusion criteria for the genetic study.

### 2.3. Clinical and Neuroradiological Data

Clinical data were retrieved from the patients’ electronic charts. The following information was collected: age at onset, gender, neurological symptoms at onset, and extra-CNS findings. Patients were divided into 3 groups according to the age at onset: (i) pediatric, 28 days–18 years; (ii) juvenile, 18–45 years; and (iii) adult, > 45 years.

Carriers and cPXE patients underwent a standard dedicated clinical workup, consisting of a thorough ophthalmological workup, slit-lamp and Goldmann visual field examination, macular optical coherence tomography, and fundus imaging with white light and autofluorescence. Further, arterial duplex ultrasounds of the carotid, vertebral arteries, and the arteries of the lower limbs were performed, as well as an echocardiography, an abdominal ultrasound, and, in males, a testicular ultrasound. In the pediatric group, brain magnetic resonance imaging (MRI) and MR angiography (MRA) were performed. In the juvenile and adult group, the neurological presentation was the reason for genetic screening and a routine neuroradiological study was performed, including brain computed tomography (CT) and CT angiography or digital subtraction angiography (DSA), when clinically indicated.

The available neuroimaging studies, including brain MRI and MRA, computed tomography angiography (CTA), and digital subtraction angiography (DSA), were reviewed in consensus by 2 neuroradiologists (AR and MS) with 25 and 15 years of experience in the pediatric subgroup and by a neuroradiologist with more than 25 years of experience (RP) as well as a vascular neurologist with dedicated training and 20 years of experience (MZ) for the juvenile and adult subgroups.

In the pediatric population, AIS was defined as the presence of an acute cerebral infarction in an arterial distribution pattern on brain imaging regardless of the presence and duration of symptoms [25]. Childhood arterial stroke was categorized according to the CASCADE criteria [26,27] and in the young and adult ages, ASCOD classification was employed [28]. Neuroradiological diagnosis of SCAD was made in the presence of at least one of the following criteria: (i) mural hematoma; (ii) dissecting pseudoaneurysm; (iii) long tapering stenosis; (iv) intimal flap; (v) double lumen or occlusion above the carotid bifurcation revealing an aneurysmal dilatation or a long tapering stenosis after recanalization in a cervical artery [29,30]. CSVD was evaluated according to the STRIVE 2.0 criteria [31].

### 2.4. Genetic Studies

Genetic analyses were performed at the Laboratory of Genetic and Genomics of Rare Diseases and Laboratory of Human Genetics of the Gaslini Children Hospital, Genoa, Italy. DNA was extracted from whole blood samples of probands and parents, purified, and quantified using standard protocols. The *ABCC6* gene was analyzed by means of diverse NGS-based strategies over the years. Hence, library preparation and successive DNA sequencing were performed on different platforms according to the different manufacturers’ instructions (Ion Ampliseq, Thermo Fisher Scientific, Waltham, Massachusetts, USA, Ion Torrent PGM Sequencer, Thermo Fisher Scientific, Waltham, Massachusetts, USA, IDT xGen Exome Research Panel v2, Illumina, San Diego, California, USA and Whole Exome Solution v2-Sophia Genetics, Rolle, Switzerland, Novaseq6000, Illumina, San Diego, California, USA). Genetic data were analyzed using specific pipelines for variant calling and filtering (custom bioinformatic pipeline developed in-house and Sophia DDM Platform-pipeline ILL1XG1G5_CNV_exome) [32,33]. Variants were filtered based on minor allele frequency (MAF) ≤2%; impact on the mature encoded protein (missense, nonsense, frameshift, splicing variants ±20 bp from the exon boundaries); in silico functional prediction considering CADD [34], Polyphen [35], and SIFT [36] tools; and/or pathogenicity classification provided by Varsome (https://varsome.com/, (accessed on 28 November 2025)) [37]. Variants were interpreted based on the consultation of different database (dbSNP, Franklin, Alamut Visual Plus, Varsome, Clinvar, LOVD, HGMD) [38,39,40] and classified according to the American College of Medical Genetics and Genomics (ACMG) criteria [41,42] and ACGS Best Practice Guidelines [ACGS Best Practice Guidelines for Variant Classification in Rare Disease 2024, v1.2 2024] [43]. Variants of uncertain significance (VUS) potentially impacting the phenotype were evaluated by a multidisciplinary team including clinicians, neuroradiologists, and geneticists in order to ascertain a possible genotype–phenotype correlation. Therefore, selected variants were validated via Sanger sequencing and evaluated according to clinical manifestations of the probands and parental segregation whenever possible.

### 2.5. Statistical Analysis

Continuous variables were summarized as means, and categorical variables were summarized as frequencies and percentages. Age differences between subjects with normal or abnormal genetic results were tested using the Mann–Whitney U test. The associations between clinical–neuroradiological and genetic findings were evaluated by the Chi-squared test and Fisher’s exact test. Statistical significance was set at *p* = 0.05. Statistical analyses were performed using SPSS Statistics software, v26 (IBM, Armonk, NY, USA).

## 3. Results

On the basis of the above-mentioned exclusion criteria, we selected 143 consecutive patients presenting with pediatric or juvenile/adult cerebral AIS or CSVD of unknown etiology with suspect genetic origin. The cohort consisted of 143 patients (77 males, 53.8%, mean age 39.3 years, range: 4 months–84 years), including 48 pediatric cases (33.6%), 24 juvenile cases (16.8%), and 71 adult cases (49.6%). Two of the pediatric cases, harboring biallelic mutations of *ABCC6*, were previously described [44]. All patients belong to the European non-Finnish ethnic group.

### 3.1. Genetic Analysis

Among the 143 selected subjects, 16 (11.2%) tested positive for a causative variant on the ABCC6 gene. The NGS sequencing platforms were designed to analyze the coding sequence and the intragenic copy-number variants. Off-target regions are therefore represented by the deep introns and the 5’ and 3’ portions of the genes. To date, no causative variants have been reported in such genomic locations (HGMD). Notably, 11 individuals (68.7%) harbored monoallelic *ABCC6* variants, while 5 patients (31.3%) carried biallelic variants. The identified variants were classified as class 4 and class 5 based on the ACMG criteria; no VUS were detected. Overall, 1/32 alleles (3.1%) carried a splicing variant, 11/32 alleles (34.4%) harbored missense variants, and 9/32 alleles (28.1%) presented nonsense/frameshift variants. Among the missense variants, 6/11 alleles (54.5%) carried the frequent hypomorphic variant c.1171A>G p.(R391G) [45,46]: five individuals presenting with a phenotype ascribable to ffPXE were heterozygous, whereas one subject suffering with GACI was compound heterozygous with another causative variant. The other identified missense changes, together with the typical loss-of-function variants, are already extensively described in the literature for their causal role. Among the entire cohort, segregation study in the family was feasible only for five patients (three ffPXE, one cPXE, one GACI). To note, for patients 14 and 15, it was not possible to ascertain the real homozygous state of the identified variants. Table 1 reports in details the genetic findings and results of the segregation studies.

### 3.2. Clinical and Neuroradiological Assessment

Patients with monoallelic variants predominantly presented in adulthood (median age at onset 54 years; range 6.7–78 years), although two pediatric cases exhibited early cerebrovascular events. The most frequent neuroradiological pattern included lacunar strokes and CSVD, observed in five adult patients. Cervical internal carotid artery (ICA) and vertebral artery (VA) dissections were also common (four patients, of which two were children), occasionally accompanied by pseudoaneurysm formation or a string-of-beads appearance. Several individuals showed intracranial stenoses, and in two cases, peri-stenotic moyamoya-like collaterals were detected. Systemic involvement was generally mild or absent: two adult patients (18.2%) exhibited renal artery involvement, one pediatric patient (20%) presented a bilateral keratoconus not directly related to cPXE or ffPXE at age 14.5 years, and the rest had no detectable extra-CNS involvement. Additionally, hypercholesterolemia and arterial hypertension were frequent comorbidities in adults but not specific to the genotype. Clinical and neuroradiological features of the subjects carrying monoallelic and biallelic *ABCC6* causative variants are summarized in Table 2.

Three of the five individuals with biallelic *ABCC6* variants presented in infancy or early childhood (onset 0.3–2.0 years). In these patients, neuroimaging revealed a diffuse and complex arteriopathy, including marked hypoplasia or aplasia of the internal carotid arteries, bilateral ICA stenosis, supraclinoid dilatation, focal vertebral artery narrowing, and moyamoya-like collateral formation. One patient exhibited a pattern similar to the one angiographically described as carotid “rete mirabile” [52,53], and another demonstrated widespread intracranial arterial calcification. The remaining two adult patients with *ABCC6* variants presented with a neuroradiological pattern of CSVD.

All children with biallelic mutations displayed significant systemic vasculopathy, including stenosis of iliac, femoral, and popliteal arteries; hypoplasia of the external iliac arteries; and infrarenal aortic tapering. Cutaneous and vascular features consistent with cPXE were present in two cases. Systemic hypertension was reported in one child. The distribution and severity of extra-CNS disease were markedly increased than in monoallelic pediatric patient, but apparently not in adult patients.

Overall, the adult cohort included 11 adult patients aged between 22 and 78 years. Commonly reported neurological symptoms among these patients included headache (patients 5, 9, 11, and 12), which in one case was a presenting symptom of carotid dissection and in the remaining three cases was part of a history of migraine. A total of 4/11 patients had a lacunar ischemic stroke in the context of a severe CSVD on brain MRI study. Two further patients had ICH as a CSVD manifestation. Only 2/11 patients had an acute dissection (1 of them with ischemic stroke treated by IV rtPA) and 1/11 patients had a TIA in the context of intracranial large artery involvement. In comparison, 6/11 patients had LV involvement, intracranial and/or extracranial, with 2 arterial dissections and 3 patients with focal bilateral arteriopathy at the ICA terminus as well as 1/11 with a corkscrew pattern on the left posterior cerebral artery, an expression of a variant of pure intracranial arteriopathy.

**Figure 1:**
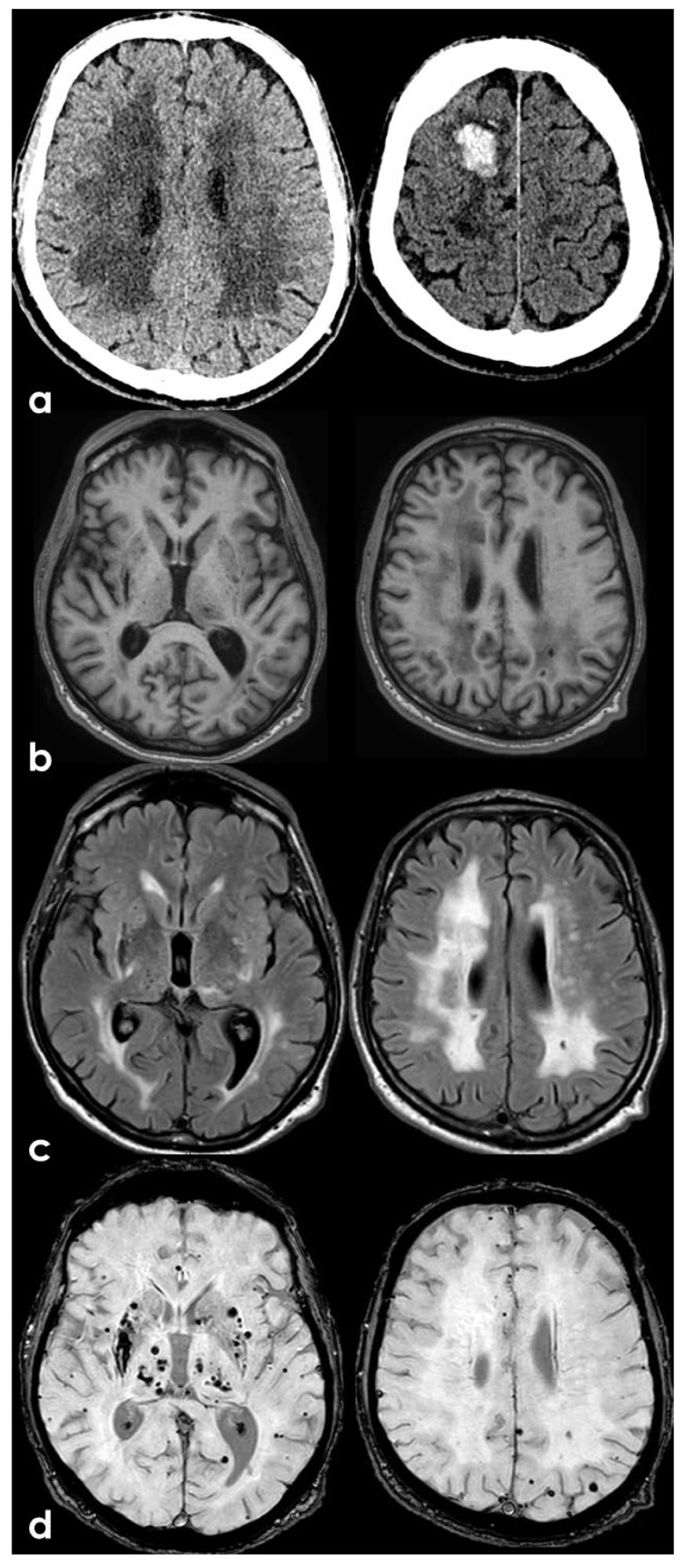
Example of CSVD associated with ABCC6 mutation in a 72-year-old woman without vascular risk factors (patient 15). Panel (**a**): non-contrast computed tomography (NCCT) of the brain showing extensive leukoaraiosis (grade 3 on the Fazekas scale) and a right frontal acute hemorrhage. Panels (**b**–**d**): brain magnetic resonance imaging (MRI) axial slices at the same two levels using different sequences [(**b**) T1W; (**c**) T2-fluid-attenuated inversion recovery—FLAIR; (**d**) susceptibility weighted imaging (SWI)], showing lacunes (**b**,**c**), leukoaraiosis (**c**), and several deep and lobar microbleeds (**d**).

**Figure 2:**
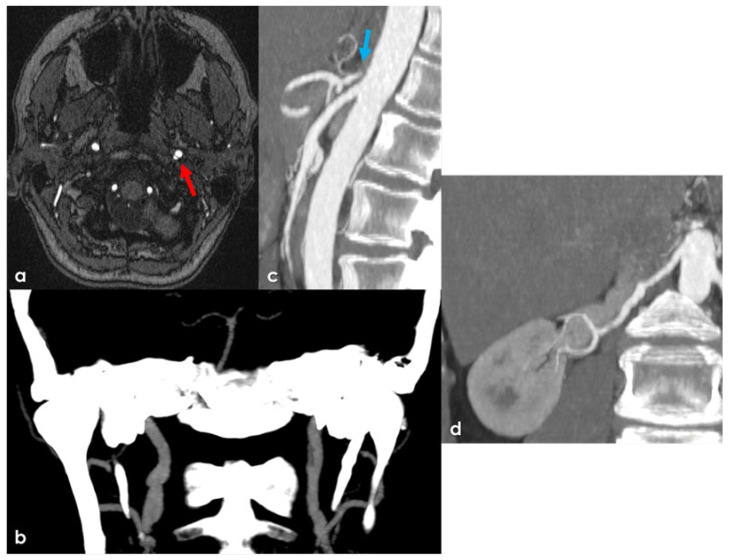
Example of a patient with LV involvement and a systemic disease (patient 7). Panel (**a**): brain magnetic resonance angiography (MRA) source image with a dual lumen in the left prepetrosal ICA (red arrow) as sign of dissection. Panel (**b**): CT angiography (CTA) processed with maximum intensity projection/multiplanar reconstruction (MIP/MPR) protocol in a coronal plane, showing the right ICA stenoses followed by vessel enlargement and the left ICA with a pseudofenestration pattern. Panel (**c**): aortic CTA (MIP/MPR in sagittal plane), showing a severe stenosis at the origin of the celiac trunk (blue arrow) and a fusiform aneurysm on the superior mesenteric artery. Panel (**d**): CTA (MIP/MPR in coronal plane) of the right renal artery, showing several irregularities in the vessel profile and a fusiform ectasia in the mid-portion.

Imaging studies in patients 7, 8, 10, 11, 13, and 15 revealed signs of CSVD (see Figure 1), characterized by acute small subcortical infarcts, white-matter hyperintensities, and cerebral microbleeds on MRI [31]. An example of LV involvement and systemic vasculopathy is reported in Figure 2.

The pediatric cohort included five patients, with ages at onset ranging from 0.3 to 9.8 years. Demographic and genetic details are summarized in Table 1 and Table 2. The most commonly reported presenting neurological symptoms were seizures (patients 12, 14, and 16), focal motor deficits such as hemiparesis (patients 1, 12, 14, and 16), and headache associated with visual or vestibular symptoms (patients 1 and 2).

**Figure 3:**
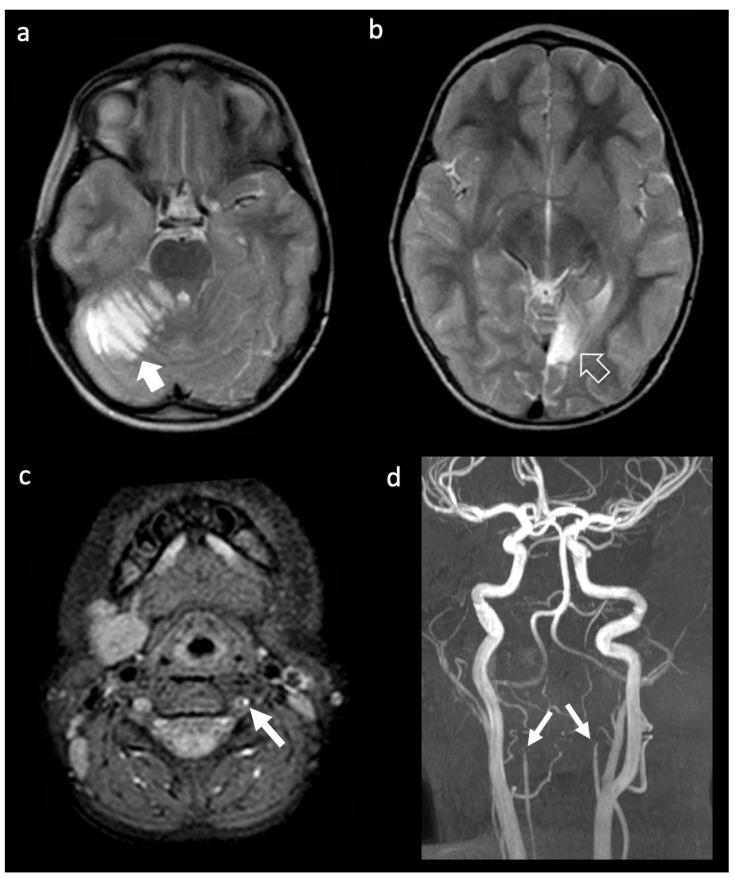
Neuroimaging findings of a pediatric patient with ffPXE (Patient 1) with a multiple AIS caused by dissection of both vertebral arteries. (**a**,**b**) Axial T2-weighted images reveal an acute right cerebellar infarct on the right (thick arrow) and a chronic occipital infarction the left (empty arrow). (**c**) Axial fat-saturated T1-weighted image demonstrates an acute hematoma within the wall of the left vertebral artery (thin arrow). (**d**) MRA, frontal view, shows bilateral interruption of the blood flow at V2-V3 level along the vertebral arteries (small thin arrows).

**Figure 4:**
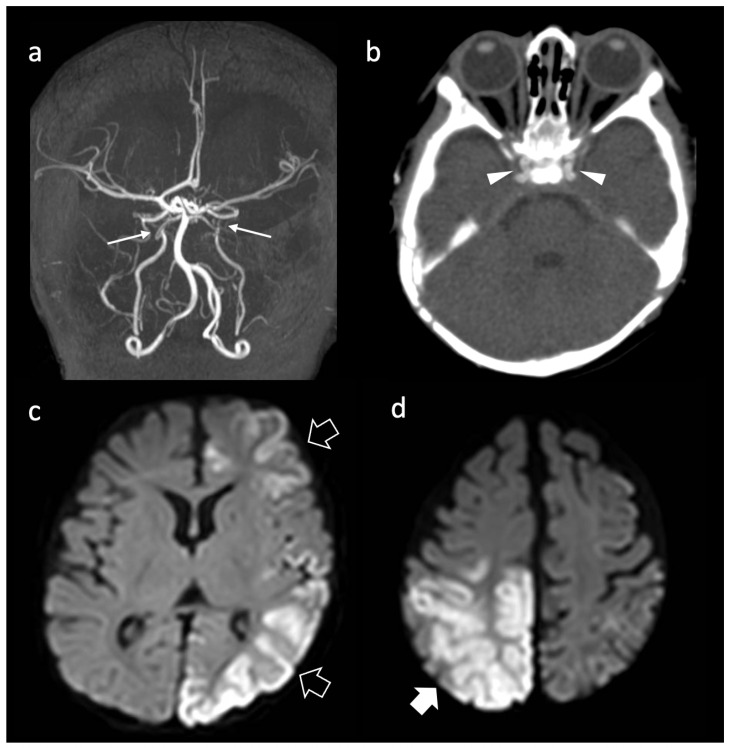
Neuroimaging features of patient 16 with GACI phenotype. (**a**) MRA, frontal view, reveals bilateral stenosis of the internal carotid arteries (thin arrows) associated with small collaterals. (**b**) Axial CT image demonstrates extensive calcifications of the affected vessels (arrowheads). (**c**,**d**) Axial diffusion-weighted images performed at clinical onset at 4 months of age (**c**) and 15 days later (**d**) demonstrate an arterial infarct in the left cerebral hemisphere (empty arrows) followed by a second arterial infarct in the right posterior frontal and parietal regions (thick arrow).

Neuroimaging demonstrated acute ischemic events in all patients, most frequently multiple asynchronous or bilateral AIS. Posterior circulation involvement due to vertebral artery dissection was observed in patients 1 and 2, with patient 2 additionally showing right PCA distal occlusion. Patients 14 and 16 presented with severe bilateral intracranial vasculopathy involving the internal carotid arteries, including intrapetrous ICA stenosis with post-stenotic fusiform dilatation (patient 14) and calcified ICA stenosis with moyamoya-like collaterals (patient 16). Patient 12 showed a distinct pattern characterized by left frontal AIS, scattered right frontal white-matter abnormalities, and marked hypoplasia/aplasia of the precervical and cervical ICAs with bilateral carotid rete mirabile [52,53]. An example of large artery involvement is illustrated in Figure 3 and Figure 4.

Extra-CNS vascular involvement was documented in four of the five patients. Patient 12 exhibited extensive systemic vasculopathy affecting the iliac arteries and the infrarenal aorta, associated with systemic hypertension. Patients 14 and 16 showed multifocal vasculopathy with stenosis of the iliac–femoral or superficial femoral and popliteal arteries, respectively, both also presenting cutaneous manifestations compatible with cPXE. Patient 2 had bilateral keratoconus during adolescence, while patient 1 displayed no extra-CNS abnormalities.

Concerning the pediatric group, a cohort study from our group [54] has already been published, including three patients (two ffPXE and one cPXE) from the present work. The long-term follow-up allowed us to clinically and neuroradiologically assess the subjects and some parents. Notably, patient 2 developed ocular bilateral keratoconus 5 years after onset, and his father, carrying the p.(R391G) variant, presented with pathognomonic PXE cutaneous manifestations on the neck. Patient 12’s mother, carrier of the p.(W967*) variant, was symptomatic for a psychiatric condition not otherwise specified and the brain MRI showed a CSVD pattern, whereas his father, carrier of the p.(K1394Nfs*9) variant, was asymptomatic and a screening brain MRI performed at 36 years of age was unremarkable.

## 4. Discussion

This study provides a detailed characterization of the cerebrovascular and systemic manifestations associated with monoallelic and biallelic *ABCC6* variants across pediatric, juvenile, and adult patients presenting with AIS- or CSVD-related manifestations. Our findings highlight a marked age-dependent divergence in phenotype expression, potentially suggesting that the impact of *ABCC6* deficiency on the vasculature is modulated by developmental stage, zygosity, and the presence of interacting genetic or environmental factors. In fact, two major cerebrovascular phenotypes emerged in our cohort: large vessel disease (LVD), predominantly affecting children but present in some adults too, and CSVD, largely confined to adults, probably requiring a longer time to develop, and able to be affected by the simultaneous effects of classic vascular risk factors. However, the presented data do not allow us to assess a direct causal relationship between *ABCC6* deficiency and CSVD and this hypothesis should be tested in larger multicohort studies.

Pediatric patients—particularly those with biallelic variants—displayed a severe and complex vasculopathy, often presenting with early-onset AIS, seizures, and focal neurological deficits. Neuroimaging frequently revealed multiple ischemic lesions in association with abnormalities of the intracranial large arteries, including intrapetrous ICA stenosis, post-stenotic fusiform dilatation, calcific arteriopathy, and moyamoya-like collateralization. Several children also exhibited developmental anomalies such as ICA hypoplasia/aplasia and a pattern similar to the one described as carotid “rete mirabile” [52,53]. If this association raises the hypothesis that *ABCC6* deficiency may interfere with normal vascular maturation and remodeling processes during early life, there are limited data to support a mechanism comparable to the one of classical neurocristopathies. Some patterns have been discussed in the literature and these are maybe more likely abnormalities than anomalies, as carotid “rete mirabile” [51], because the embryological existence of a rete-like organization of extra- and intracranial large vessels has been questioned as not functional and not needed in humans. It is possible that some of these patterns derive from a very early hit on genetically frail vessels, then combining a genetic predisposition with an acquired source of damage and a further evolution of the arteriopathy during natural history of the disease.

In contrast, adult carriers typically exhibited a predominantly microvascular phenotype, with lacunar ischemic strokes and classical CSVD signatures—including small subcortical infarcts, white-matter hyperintensities, and microbleeds—being the most prominent neuroradiological features, as standardized for clinical and research descriptions [31]. However, among the spectrum of manifestations related with *ABCC6* gene mutations, several cerebrovascular patterns have been described, in particular large artery involvement with an increased risk of spontaneous carotid and vertebral dissection from one side and CSVD signatures on the other side. In some cases, both patterns of cerebrovascular involvement can be present in the same individual. Both large-vessel and small-vessel involvement may be symptomatic for acute ischemic stroke or not. As previously considered, CSVD pattern seems to be present in adult cohorts and not in the pediatric population, although the design of the study does not allow to establish a direct causality link. All the patients with CSVD but one carried a monoallelic *ABCC6* variant, whereas LVD subjects were either heterozygous or compound heterozygous. The presence of small subcortical infarcts, white-matter hyperintensities, and cerebral microbleeds aligns with the understanding that *ABCC6* mutations can contribute to microvascular damage [12,19]. In adults, vascular calcifications are not the main hallmark of the disease, maybe because of the common finding of intracranial artery calcification with increasing age. However, a recent study by Bartstra et al. [55] demonstrated that patients with cPXE exhibit significantly higher arterial pulsatility (1.05) compared to controls (0.94), suggesting that carotid siphon calcification contributes to microvascular damage and subsequent ischemic events. Classical vascular risk factors can contribute to the development of cerebrovascular disease in these patients. A notable proportion of patients (67%) presented with hypertension, a critical risk factor for cerebrovascular disease, in particular for CSVD and its acute manifestations. In our adult cohort, all but one patient had arterial hypertension and all but one had hypercholesterolemia. In addition, dyslipidemic profiles were observed in multiple patients, correlating with increased cerebrovascular risk [12]. These findings reflect the systemic implications of *ABCC6* deficiency, where the interplay of genetic and environmental factors exacerbates cerebrovascular risks.

In addition to neurological symptoms and neuroradiological findings, patients exhibited various extra-CNS features both in the risk profile and in systemic arteries. A systemic arteriopathy was present in only 2/11 patients in the adult cohort, highlighting the different profile of adult vs. pediatric patients.

As mentioned, cPXE and GACI patients showed a marked intra- and extracranial vasculopathy. One hypothesis is that *ABCC6* deficit may interfere with vascular maturation or remodeling processes that are most active in early life. The presence of congenital or developmental abnormalities—such as ICA hypoplasia/aplasia—in several pediatric cases further supports the notion that the impact of *ABCC6* variants may be particularly relevant during cerebrovascular development, rather than solely through degenerative mechanisms. Another possibility is that the lack of *ABCC6* functional protein may facilitate the expression of vascular damage with concurrent hits early in life, starting from the prenatal period. Conversely, adult biallelic patients are not consistently different from the monoallelic ones in terms of neuroradiologic involvement, which is mainly the CSVD pattern.

Extra-CNS findings strengthened this age-related distinction. While systemic vasculopathy and cutaneous features were common among pediatric patients, they were poorly represented in adults. The widespread involvement of iliac, femoral, and popliteal arteries in children raises the possibility that reduced *ABCC6* function—through impaired ATP/PPi metabolism and consequent dysregulation of ectopic mineralization—may have a stronger impact during periods of rapid vascular growth.

In both the cohorts, the zygosity of the diverse variants arose in distinct phenotype consistent with ffPXE, cPXE, and GACI. As described in the literature, there is no genotype–phenotype correlation related to the type of variant [47,48,49,50,51]. The mechanisms connecting ABCC6 mutations to increased stroke risk are multifaceted and discordant over the years. Due to the high frequency of carriers of pathogenic *ABCC6* in the general population (~1%), its causative role has been largely debated [14,17,56]. However, De Vilder et al. [12] demonstrated that pathogenic *ABCC6* variants were 4.9 times more frequent in an independent adult ischemic stroke cohort compared with healthy controls. Subsequently and anecdotally, other authors described monosymptomatic forms, ascribable to ffPXE, in an adult cohort including intracranial or systemic vascular diseases, ocular disorders, and cutaneous manifestations [10,51,57], hypothesizing that it could be due to hypothetical haploinsufficiency [7,9]. All the *ABCC6* variants identified in this study are already described in the literature and their causal role has been extensively documented. The effect of the frequent hypomorphic variant c.1171A>G p.(R391G) has been largely debated as pathogenic [45,46], being frequent in the European non-Finnish population (0.79%) and having a low penetrance (2%) [49]. However, a number of cPXE and GACI patients harbor this variant in trans with a causative one, leading to the hypothesis that such a variant can become fully penetrant when other interacting protein in the biochemical pathway of the *ABCC6* gene are not properly functional. In light of this, considering the genetic data from this pediatric/juvenile/adult stroke cohort, we might suppose that these findings are also transposable to ffPXE patients.

According to Zedde and Pascarella [20], *ABCC6* is crucial for regulating ectopic calcification and modulating extracellular purinergic pathways. Defective *ABCC6* leads to decreased levels of inorganic pyrophosphate (PPi), a vital inhibitor of calcification, resulting in vascular calcifications that mimic atherosclerotic changes [58]. Studies indicate that *ABCC6* deficiency contributes to dyslipidemia, characterized by decreased HDL and increased LDL levels, ultimately promoting atherosclerosis [59]. This atherosclerotic process is significant given that ischemic strokes are often a consequence of large vessel disease [12]. Immunostaining studies in *ABCC6*-deficient mice revealed dysregulation of BMP and TGFβ signaling pathways, suggesting a pro-ischemic state that may lower the threshold for acute ischemic events [12]. Although there is no apparent correlation between PPi plasma levels and *ABCC6* genotype and clinical phenotype, it has been functionally measured that PPi plasma levels are decreased in cPXE patients and even in carriers compared to controls [1]. This demonstrates an effect of the monoallelic variants in impairing the biological processes regulated by the *ABCC6* protein. This aligns with findings from Bartstra et al. [52], emphasizing the need for molecular analyses of *ABCC6* in patients with cryptogenic strokes presenting as CSVD or LVD with vasculopathy. However, adult CSVD findings may reflect gene/environment interaction rather than direct genetic causation.

The study is limited by the relatively small sample size and the inherent heterogeneity of clinical presentations. Imaging protocols were not fully standardized across age groups, and long-term longitudinal data were incomplete for some participants. Genetic analyses did not include comprehensive exploration of modifying genes that may influence the penetrance of the *ABCC6*-related manifestations. A major limitation of this study is the absence of a control group (healthy controls matched by age and sex and stroke patients without ABCC6 variants). This issue does not allow us to definitively establish whether monoallelic *ABCC6* variants represent an independent CSVD driver or a component of a wider susceptibility background. In fact, the lack of a control group for adult patients may have led to an overestimation of the prevalence of the CSVD phenotype, despite the paucity of concomitant vascular risk factors to justify this pattern.

Future research should focus on the following:

- Longitudinal studies assessing cerebrovascular health over time in patients with *ABCC6* mutations.
- Investigating the broader genetic landscape related to *ABCC6* and the interplay with other genetic factors implicated in vascular health [60].

## 5. Conclusions

Our findings underscore the importance of considering *ABCC6* variants in both pediatric and adult patients presenting with cryptogenic AIS, CSVD, or atypical vasculopathy. Identifying these mutations may enhance risk stratification and inform management strategies, particularly concerning traditional vascular risk factors such as hypertension and dyslipidemia, especially in adults, given the synergistic effect of hypertension and dyslipidemia on *ABCC6*-related vascular fragility. In addition, *ABCC6* heterozygosity may contribute to CSVD phenotype, acting as a vulnerability or modifier factor, especially within a multifactorial background in the presence of conventional vascular risks.

A deep understanding of the mechanisms linking these mutations to cerebrovascular complications in *ABCC6*-related diseases will be vital for developing targeted therapeutic strategies.

## Figures and Tables

**Table 1:** *ABCC6* variants identified in the present cohort.

Pts ID	Variant Description								
	cDNA	Protein	Segregation	ACMG Class	dbSNP	Clinvar ID	MAF (NFE)	Homozygotes	Allele Ref
PXE*-ff* PATIENTS									
1	c.2096A>G	p.(E699G)	maternal	5	rs1252723064	1445010	0.0017%	0%	[47]
2	c.1171A>G	p.(R391G)	paternal	4	rs72653762	265017	0.79%	0.003%	[48,49]
3	c.3736-1G>A	p.(?)	paternal	5	rs63750273	6574	0.008%	0%	[47]
4	c.1171A>G	p.(R391G)	NA	4	rs72653762	265017	0.79%	0.003%	[48,49]
5	c.2504G>A	p.(G835D)	NA	5	rs199990104	-	0.002%	0%	[48]
6	c.1171A>G	p.(R391G)	NA	4	rs72653762	265017	0.79%	0.003%	[48,49]
7	c.1171A>G	p.(R391G)	NA	4	rs72653762	265017	0.79%	0.003%	[48,49]
8	c.1171A>G	p.(R391G)	NA	4	rs72653762	265017	0.79%	0.003%	[48,49]
9	c.4070G>A	p.(R1357Q)	NA	5	rs201275608	1359254	0.008%	0%	[50]
10	c.4182del	p.(K1394Nfs*9)	NA	5	rs67791546	433351	0%	0%	[47]
11	c.3421C>T	p.(R1141*)	NA	5	rs72653706	6559	0.22%	0%	[47,49]
**CLASSICAL PXE PATIENTS**									
12	c.4182del	p.(K1394Nfs*9)	paternal	5	rs67791546	433351	0%	0%	[47]
	c.2900G>A	p.(W967*)	maternal	5	rs2046997344	872925	-	-	[48]
13	c.3421C>T	p.(R1141*)	NA	5	rs72653706	6559	0.22%	0%	[47,49]
	c.3774dup	p.(W1259Lfs*19)	NA	5	rs72664220	433330	0.0038%	0%	[48]
14	c.3421C>T	p.(R1141*)	NA	5	rs72653706	6559	0.22%	0%	[47,49]
	c.3421C>T	p.(R1141*)	NA						
15	c.3413G>A	p.(R1138Q)	NA	5	rs60791294	6561	0.01%	0%	[51]
	c.3413G>A	p.(R1138Q)	NA						
**GACI PATIENT**									
15	c.1552C>T	p.(R518*)	paternal	5	rs72650700	30339	0.0079%	0%	[47,48]
	c.1171A>G	p.(R391G)	maternal	4	rs72653762	265017	0.79%	0.003%	[48,49]

Legend: all the variants listed refer to the ABCC6 gene (NM_001171.6); MAF refers to the minor allele frequency in the non-Finnish European ethnic group; NA = not available.

**Table 2:** Genetic, clinical, and neuroradiological features of *ABCC6* heterozygous carriers and cPXE and GACI cases.

pts ID	ABCC6 Variant	ABCC6 Phenotype	Gender	Risk Factor	Onset Age (years)	Minor Trauma	Clinical Features at Onset							Neuroradiological Features at Onset	Extra-CNS Findings
							HS	VD	LD	HA	HEM	S	V		
1	p.(E699G)	ffPXE	M	No	6	yes	-	+	+	+	+	-	-	multiple asynchronous AIS, bilateral VA dissection	No
2	p.(R391G)	ffPXE	M	No	9	yes	-	-	-	+	+	-	+	multiple asynchronous AIS, r-VA dissection, r-PCA distal occlusion	bilateral keratoconus (14y)
3	c.3736-1G>A	ffPXE	M	*, $	44	no	+	-	+	+	+	-	-	l-ICA dissection, vessel occlusion, AIS	No
4	p.(R391G)	ffPXE	F	*, $, %	64	no	-	-	+	+	+	-	-	bilateral arteriopathy, moyamoya-like pattern	No
5	p.(G835D)	ffPXE	F	*, $	66	no	-	-	+	+	+	-	-	CSVD	No
6	p.(R391G)	ffPXE	F	*, $	78	no	-	-	+	+	+	-	-	CSVD	No
7	p.(R391G)	ffPXE	F	*, $	49	no	-	-	-	+	-	-	-	l-ICA dissection, pseudoaneurysm, carotid and vertebral arteries string-of-beads	r-RA focal ectasia, celiac trunk stenosis, MA aneurysm
8	p.(R391G)	ffPXE	F	*, $	58	no	-	-	+	+	+	-	-	CSVD, r-VA stenosis, ectasia	No
9	p.(R1357Q)	ffPXE	F	*, $	54	no	-	-	+	+	+	-	-	CSVD	No
10	p.(K1394Nfs*9)	ffPXE	F	*, $	22	no	-	-	-	+	-	-	-	bilateral arteriopathy	bilateral RA stenosis
11	p.(R1141*)	ffPXE	M	*, $	44	no	+	-	+	+	+	-	-	CSVD	No
12	p.(K1394Nfs*9) p.(W967*)	cPXE	M	No	2	no	-	-	-	-	+	+	-	AIS, scattered WM signal alterations, ICA hypoplasia/aplasia, bilateral carotid rete mirabile	multifocal vasculopathy, systemic hypertension
13	p.(R1141*) p.(W1259Lfs*19)	cPXE	M	*, $	67	no	-	-	+	+	+	-	-	CSVD	No
14	p.(R1141*) p.(R1141*)	cPXE	F	No	0.4	no	-	-	-	-	+	+	-	multiple bilateral AIS, bilateral stenosis ICAs, post-stenotic fusiform dilatation, focal narrowing of l-VA	multifocal vasculopathy, skin PXE signs
15	p.(R1138Q) p.(R1138Q)	cPXE	F	No	72	no	-	-	-	-	+	-	-	CSVD and macrobleeds	No
16	p.(R518*) p.(R391G)	GACI	M	No	0.3	no	-	-	-	-	+	+	-	multiple bilateral AIS, bilateral stenosis of ICAs with calcifications, moyamoya-like collaterals	multifocal vasculopathy, arterial calcifications, skin PXE signs

Legend: ffPXE = pseudoxanthoma elasticum–forme fruste, cPXE = classical pseudoxanthoma elasticum, GACI = generalized artery calcification of infancy, * = hypercholesterolemia, $ = arterial hypertension, % = diabetes, HS = Horner syndrome, VD = visual deficit, LD = language disorder, HA = headache, HEM = hemiparesis, S = seizures, V = vomit, AIS = arterial ischemic stroke, CSVD = cerebral small vessel disease, ICA = intracranial carotid artery, PCA = posterior carotid artery, VA = vertebral artery, RA = renal artery, MA = mesenteric artery, WM = white matter.

## Data Availability

The data generated in this investigation are anonymized and available upon reasonable request.

## Abbreviations

The following abbreviations are used in this manuscript:

CNS	Central Nervous System
ICH	Intracerebral Hemorrhage
MRI	Magnetic Resonance Imaging
CSVD	Cerebral Small Vessel Disease
